# Effects of sex and birth weight on non-specific health services use following whole-cell pertussis vaccination: a self-controlled case series analysis

**DOI:** 10.1080/21645515.2019.1586029

**Published:** 2019-04-05

**Authors:** Steven Hawken, Robin Ducharme, Deshayne B. Fell, Assaf P. Oron, Kumanan Wilson

**Affiliations:** a Clinical Epidemiology Program, Ottawa Hospital Research Institute, Ottawa, Ontario, Canada; b School of Epidemiology and Public Health, University of Ottawa, Ottawa, Canada; c ICES, University of Ottawa, Ottawa, Canada; d Children’s Hospital of Eastern Ontario (CHEO) Research Institute, Children’s Hospital of Eastern Ontario (CHEO), Ottawa, Ontario, Canada; e Maternal, Newborn, and Child Health, Institute for Disease Modeling, Bellevue, Washington, USA; f Department of Medicine, University of Ottawa, Ottawa, Ontario, Canada

**Keywords:** Vaccine, self-controlled case series, pediatrics, whole-cell pertussis

## Abstract

Previous studies from low-resource countries have highlighted concerns surrounding non-specific effects of whole-cell pertussis vaccination, particularly in females. We sought to examine the effects of sex and birth weight on health services utilization following first exposure to whole-cell pertussis vaccine. Using a self-controlled case series design and by calculating relative incidence ratios (RIRs), we compared the relative incidence of emergency department visits and/or hospital admissions between sexes and between birth weight quintiles. Females had a higher relative incidence of events following vaccination compared to males (RIR = 1.13, 95% CI: 0.99, 1.30), which persisted after adjustment for birth weight (RIR = 1.12, 95% CI: 0.97, 1.28). We also observed a trend of increasing relative incidence of events over decreasing quintiles of birth weight; infants in the lowest quintile had a 26% higher relative event rate compared to the highest quintile, which was robust to adjustment for sex (Unadjusted RIR = 1.26, 95% CI: 1.01, 1.56; Adjusted RIR = 1.23, 95% CI: 0.99, 1.53). The risk of all-cause health services utilization immediately following vaccination, was elevated in female infants and infants having lower birth weight. Further study is warranted to determine if vaccine dosing should take infant weight into account.

## Introduction

Immunization is widely accepted as one of the most beneficial interventions in population health. While today’s vaccines are rigorously tested for safety and efficacy, there remains some uncertainty with respect to non-specific effects of vaccination, which are separate from the intended effect: protection against infectious disease. One routinely-administered childhood vaccine is designed to protect against pertussis, a highly contagious bacterial infection, and an important cause of infant illness and deaths worldwide. ^1^ In Canada, a whole-cell pertussis vaccine was introduced in 1943, and led to a substantial decrease in pertussis incidence and associated morbidity and mortality.^2^ However, concerns surfaced over the whole-cell vaccine’s reactogenicity following reports of adverse reactions. ^3-5^ Although this vaccine was eventually replaced with an acellular version across Canadian provinces and territories between July 1997 and April 1998, ^6^ the whole-cell formulation remains widely used in low-resource countries. ^7^ Some observational studies from low-resource countries have reported non-specific vaccine effects including an increase in mortality, with potential effect modification by gender. However, a World Health Organization commissioned review found no randomized clinical trials examining this question for the pertussis vaccine. The combined evidence from observational studies was deemed inconclusive, despite pointing on average towards increased mortality. ^8-10^

In view of the low infant mortality rate in Canada, non-specific vaccine effects can be studied by examining health services utilization following vaccination as a proxy for general reactogenicity. In a series of studies in the province of Ontario, we have previously demonstrated an increase in all-cause health services utilization in the first 3 days following whole-cell Diphtheria, Tetanus, and Pertussis vaccination compared to the acellular version, ^11^ as well as an increase in all-cause health services use in females in days 4 to 12 following the 12-month Measles, Mumps and Rubella vaccine. ^12^ Moreover, we have also found that birth weight, independent of preterm birth, has an important effect on the risk of health services use following exposure to the acellular pertussis vaccine at 2 months of age. ^13^ In this study, we sought to examine the effect of sex on all-cause health services utilization during the years in which the whole-cell pertussis vaccine was administered using population-based health administrative data. We hypothesized that the sex difference in risk of non-specific vaccine effects observed in previous studies may be partly mediated by birth weight. We investigated this hypothesis by analyzing the effects of both sex and birth weight on health services use following the 2-month whole-cell pertussis vaccination.

## Results

### Effect by sex

**Figure 1.**
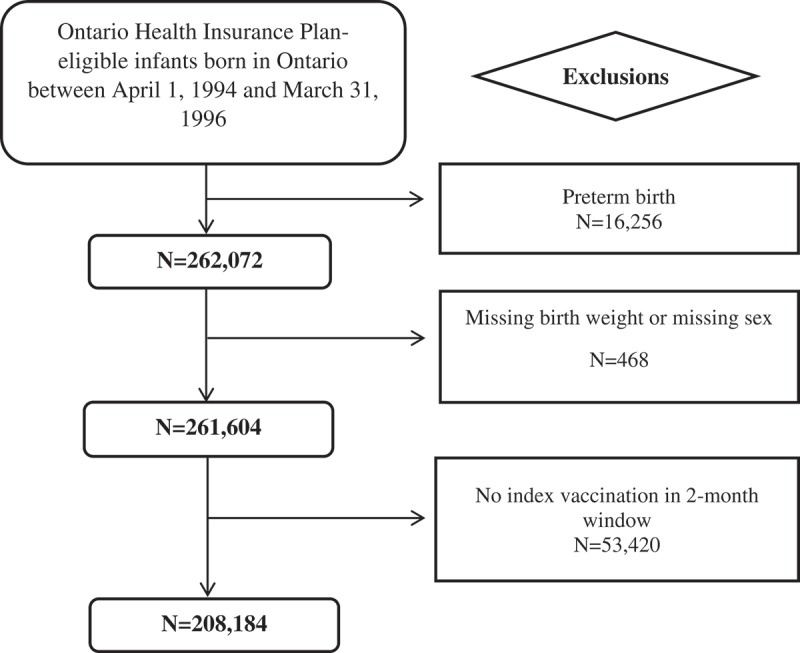
Study cohort creation diagram.

Our analysis included data on 208,184 children born between April 1, 1994 and March 31, 1996 who received the whole-cell pertussis vaccine at 2 months of age (Figure 1). The day-to-day variation in incidence of events in males and females relative to the date of vaccination is illustrated in Supplementary Figure 1. Relative incidence and relative incidence ratios are based on pooled incidences on the days contributing to the risk and control periods. In female infants, the unadjusted relative incidence in the risk vs. control period was significantly greater than 1 (RI = 1.15, 95% CI 1.04, 1.27) compared to 1.02 (0.93, 1.11) in males. This yielded an unadjusted relative incidence ratio comparing females to males of 1.13 (95% CI 0.99, 1.30), which translates to 69 excess events per 100,000 vaccinated females compared to the number of events that would have occurred in the same number of vaccinated males. This increase persisted after adjustment for birth weight.

### Effect by birth weight

Supplementary Figure 2 graphically displays the frequency of events on each day relative to the date of vaccination by birth weight quintile. Among the infants with the lowest birth weight (quintile 5), the incidence of events in the first 3 days following vaccination was significantly higher than in the control period in both the unadjusted model, and in the model adjusted for birth weight (Table 1). We observed a pattern of increasing relative incidence with decreasing quintile of birth weight which approached statistical significance, with the lowest birth weight infants having a 25% relative increase in event rate compared to the largest infants, and this persisted after adjustment for sex. The unadjusted relative incidence ratio (95% CI) comparing infants in the lowest birth weight quintile to infants in the highest quintile was 1.26 (1.01, 1.56), which translates to 130 excess events per 100,000 vaccinated low birth weight infants compared to the number of events that would have occurred in the same number of vaccinated high birth weight infants.

**Table 1.** Emergency department visits and/or hospitalization following 2-month vaccination, by sex and birth weight quintiles.

Sex	Vaccinated Children	Events During Risk Period (Days 0–2)	Events During Control Period (Days 9–18)	Relative Incidence of Events (95% CI) Unadjusted	Relative Incidence of Events (95% CI) Adjusted for bweight	Unadjusted Relative Incidence Ratio (95% CI), *P* value	Adjusted Relative Incidence Ratio (95% CI), *P* value
Male	106674	609	1798	1.02 (0.93, 1.11)	1.52 (0.93, 2.48)	1 (ref)	1 (ref)
Female	101510	540	1407	1.15 (1.04, 1.27)	1.70 (1.06, 2.72)	1.13 (0.99, 1.30), p = 0.0701	1.12 (0.97, 1.28), p = 0.1195
Birth Weight Quintile	Vaccinated Children	Events During Risk Period (Days 0–2)	Events During Control Period (Days 9–18)	Relative Incidence of Events (95% CI) Unadjusted	Relative Incidence of Events (95% CI) Adjusted for sex	Unadjusted Relative Incidence Ratio (95% CI), *P* value^a^	Adjusted Relative Incidence Ratio (95% CI), *P* value^b^
Q1: ≥3871g	40724	185	558	0.99 (0.84, 1.17)	0.96 (0.81, 1.14)	1 (ref)	1 (ref)
Q2: 3581-3870g	41031	208	636	0.98 (0.84, 1.15)	0.94 (0.80, 1.11)	0.99 (0.78, 1.24), p = 0.9067	0.98 (0.78, 1.23), p = 0.8653
Q3: 3341-3580g	41596	217	622	1.05 (0.90, 1.22)	1.00 (0.84, 1.18)	1.05 (0.84, 1.32), p = 0.6599	1.04 (0.83, 1.30), p = 0.7421
Q4: 3063-3340g	43129	245	683	1.08 (0.93, 1.24)	1.02 (0.87, 1.20)	1.08 (0.87, 1.35), p = 0.4853	1.06 (0.85, 1.33), p = 0.5780
Q5: ≤3062g	41704	294	706	1.25 (1.09, 1.43)	1.18 (1.01, 1.38)	1.26 (1.01, 1.56), p = 0.0375	1.23 (0.99, 1.53), p = 0.0610

^a^ *P* value for overall interaction between birth weight and risk period (unadjusted model) = 0.1386

^b^ *P* value for overall interaction between birth weight and risk period (model adjusted for sex) = 0.2048

**Table 2.** Hospitalization following 2-month vaccination, by sex and birth weight quintiles.

Sex	Vaccinated Children	Events During Risk Period (Days 0–2)	Events During Control Period (Days 9–18)	Relative Incidence of Events (95% CI) Unadjusted	Relative Incidence of Events (95% CI) Adjusted for bweight	Unadjusted Relative Incidence Ratio (95% CI), *P* value	Adjusted Relative Incidence Ratio (95% CI), *P* value
Male	106674	84	440	0.57 (0.45, 0.72)	0.99 (0.29, 3.45)	1 (ref)	1 (ref)
Female	101510	71	295	0.72 (0.56, 0.94)	1.21 (0.37, 3.96)	1.26 (0.89, 1.79), p = 0.1929	1.22 (0.86,1.74), p = 0.2702
Birth Weight Quintile	Vaccinated Children	Events During Risk Period (Days 0–2)	Events During Control Period (Days 9–18)	Relative Incidence of Events (95% CI) Unadjusted	Relative Incidence of Events (95% CI) Adjusted for sex	Unadjusted Relative Incidence Ratio (95% CI), *P* value^a^	Adjusted Relative Incidence Ratio (95% CI), *P* value^b^
Q1: ≥3871g	40724	25	106	0.71 (0.46, 1.09)	0.67 (0.43, 1.05)	1 (ref)	1 (ref)
Q2: 3581-3870g	41031	20	128	0.47 (0.29, 0.75)	0.44 (0.27, 0.71)	0.66 (0.35, 1.26), p = 0.2087	0.65 (0.34, 1.24), p = 0.1925
Q3: 3341-3580g	41596	25	147	0.51 (0.33, 0.78)	0.47 (0.30, 0.74)	0.72 (0.39, 1.32), p = 0.2919	0.70 (0.38, 1.29), p = 0.2500
Q4: 3063-3340g	43129	35	169	0.62 (0.43, 0.89)	0.57 (0.38, 0.85)	0.88 (0.50, 1.55), p = 0.6536	0.84 (0.48, 1.50), p = 0.5613
Q5: ≤3062g	41704	50	185	0.81 (0.59, 1.11)	0.73 (0.50, 1.05)	1.15 (0.67, 1.96), p = 0.6185	1.08 (0.63, 1.87), p = 0.7764

^a^ *P* value for overall interaction between birth weight and risk period (unadjusted model) = 0.2668

^b^ *P* value for overall interaction between birth weight and risk period (model adjusted for sex) = 0.3171

### Sensitivity analyses

Most of the observed events were ED visits (~80%). When examining hospital admissions alone, there was evidence of a similar pattern of increased relative incidence in female infants, and in those with the lowest birth weight, as was seen for ED visits and hospital admissions combined. However, these results were not statistically significant (Table 2).

Although 44% of the hospital admissions and ED visits observed in the risk period occurred within the first 24 hours following vaccination, the sex difference in relative incidence of events was only apparent when including events within 72 hours of vaccination in the analysis (Supplementary Table 1). Similar to our 2-month analysis, our 4- and 6-month analyses showed an increased relative incidence of events in females compared to males, but with attenuation of the RIR observed for the 2-month vaccination.

In a post-hoc sensitivity analysis, we compared the relative incidence of events between infants having a birth weight <2500g and infants weighing 2500g or more at birth. There was no significant difference in relative incidence of events between the two groups in the unadjusted analysis, or in the analysis adjusted for sex (Unadjusted RIR = 0.94, 95% CI = 0.61–1.45; Adjusted RIR = 0.92, 95% CI = 0.60–1.42).

## Discussion

In this study, we demonstrate an increased risk of all-cause admissions and ED visits in female infants, immediately after the first exposure to the whole-cell pertussis vaccine at 2 months of age, compared to a control period. Although not statistically significant, there is a pattern of higher relative incidence of events in females compared to males. These findings persist after adjustment for birth weight, indicating that the sex effect is relatively independent of birth weight. We also demonstrate that infants in the lowest birth weight quintile have an increased relative incidence of events immediately following vaccination compared to the control period, and this relative incidence is trending toward a significant increase compared to the highest birth weight quintile, after adjustment for sex. Our post-hoc sensitivity analysis comparing infants born at <2500g vs ≥2500g found no significant difference in relative incidence of events between the two groups. The absence of a significant effect in this analysis is likely due to infants under 2500g being a highly select population with a high baseline rate of events, making it difficult to discern a signal from the reactogenicity of a vaccine. Taken together, our findings suggest there are independent effects of sex and, in particular, birth weight on the risk of admissions and ED visits following whole-cell pertussis vaccination at 2 months of age.

Several other studies have found evidence of an increased risk of non-specific vaccine effects in females compared to males. Similar to our results, the World Health Organization review of studies in low-resource countries found some evidence for differential increased mortality among girls, but the evidence was not significant. ^8^ One should note that the observation period in those studies lasted several months to over a year, thus including both our observation period of 0–72 hours post-vaccination, as well as our control period of 9 to 18 days post-vaccination. Therefore, it is unclear whether the effects observed in our high-resource, low-mortality and intensively monitored settings share the same potential etiology as the longer-term effects reported in the less closely monitored, low-resource and high-mortality settings. Our results are consistent with an earlier study conducted by members of our group that identified an increased risk of health services utilization with decreasing birth weight in term infants following the 2-month acellular pertussis vaccination. ^13^ Interestingly, our previous analysis of the impact of sex on the risk of events following the 2-month vaccination did not identify any effect with the acellular pertussis vaccine, ^12^ which is known to have a considerably lower reactogenicity profile compared with the whole cell pertussis vaccine.

Our use of birth weight as a proxy for weight at the time of vaccination at 2 months of age is based on the strong correlation between birth weight and infant weight at 2 months. ^14^ We hypothesize that the observed effect of birth weight on risk of health services utilization following immunization could be partly attributable to the fact that the same dose of vaccine is administered to all infants regardless of weight, resulting in lower birth weight infants receiving more vaccine per unit of body weight as compared to higher birth weight infants. An animal study that found the risk of vaccine adverse events to be correlated with body weight supports this hypothesis. ^15^ Moreover, body weight is taken into account for dosing of other pharmaceuticals given to infants and children. ^16^

Many studies have recognized the important physiological differences between sexes that affect their immunologic responses, ^17-20^ including those related to hormones levels. Variations in immune responses to vaccines by sex have been reported, both in immunogenicity^19,21^ and vaccine reactogenicity following the live-attenuated rubella, ^22^ and the high- and standard-titre measles vaccines. ^23-25^ Given this evidence, there may be a physiological and immunological basis to the sex effect we observed with the whole-cell pertussis vaccine in this study. We also hypothesized that the sex difference was partially mediated by birth weight, but since the effect of sex was robust to adjustment by birth weight, it appears that there are independent effects of both variables on the risk of health services use following whole-cell pertussis vaccination.

Strengths of our study include the examination of the impact of birth weight in term infants, the large sample size and the use of the self-controlled case series design, which allowed us to adjust for fixed confounders. By using a case-only design, where cases serve as their own controls, this design can reduce the impact of selection bias and unequal distribution of confounding variables between vaccinated and unvaccinated infants. This is particularly important in studying population-wide vaccinations as vaccinated infants are fundamentally different from unvaccinated infants. This is one of the reasons that the SCCS is a widely adopted study design in studying adverse events following vaccination. ^26,27^ The use of RIRs to compare relative incidences of events between sexes allows us to adjust for confounding such as the healthy vaccinee effect. On the other hand, the use of RIRs can introduce other potential confounding as the resulting effect estimates are no longer within an individual. ^28^ We have taken steps to mediate this by adjusting for sex in the analysis of birth weight, and by adjusting for birth weight in the analysis of sex, but we cannot exclude the possibility of unmeasured confounding impacting our results. The use of all-cause health services utilization as an outcome represents both a strength and a weakness of our study. The use of all-cause events allowed us to capture non-specific vaccine effects, including events related to immunomodulation. The 5 most common diagnoses for the events, based on diagnosis codes, were: dyspnea and respiratory abnormalities, acute bronchiolitis, other and unspecified complications of medical care, fever and convulsions. On the other hand, less severe events that do not result in an ED visit would be missed.

One limitation of the data is that only general vaccination codes were available. While we cannot be certain that the vaccination administered at 2 months of age was pertussis vaccine, we are confident in this assumption given the schedule of Ontario’s publicly-funded immunization program. As pertussis vaccine is administered as part of a combination vaccine, our study is unable to ascertain with certainty whether the effects we observed are due to pertussis or other components of the vaccine. However, previous studies have demonstrated that the whole cell pertussis component of the vaccine is the most reactogenic as the switch to acellular pertussis considerably reduced the adverse event profile.

Further, previous studies have found that the risk of non-specific vaccine effects is altered by the sequence in which vaccines are given, which we could not address using general vaccination codes. ^10^ In our analysis we assume that the risk and control periods are consistent between males and females. Our analysis of RIRs further assumed that the healthy vaccinee effect manifested similarly in the compared subgroup (sex and birth weight). Conversely, studies that report RIs without attempting to account for the healthy vaccinee effect have likely underestimated effect sizes. ^28^ A limitation of all self-controlled case series analyses is the possibility of coincident temporal exposures; however, to our knowledge there are no other routinely-administered interventions at 2 months of age.

Finally, our use of birth weight as a proxy for body weight at the time of the 2-month vaccination may have introduced measurement error due to imperfect correlation between birth weight and body weight at 2 months of age. However, this likely would have biased observed differences in relative incidences according to birth weight towards the null.

In conclusion, we observed an increased risk of all-cause health services utilization following whole-cell pertussis vaccination in the first 3 days following immunization, in female vs. male infants, and in infants having a low birth weight. In a previous study conducted during the period in which the acellular Diphtheria, Tetanus, and Pertussis vaccine was used, we did not find any effect of sex following the 2-month vaccination. Further study is needed to explore the biological mechanisms surrounding this observed sex difference associated with the whole-cell version. Moreover, additional study is warranted to determine if vaccine dosing should take infant weight into account.

## Methods

### Data

We conducted this study using VISION (Vaccine and Immunization Surveillance in Ontario), an analysis platform that was created using linked health administrative data to monitor vaccine safety and effectiveness in Ontario, Canada. ^29^ Using this infrastructure, we examined the effects of sex and birth weight on rates of emergency department (ED) visits and/or hospital admissions within pre-defined risk periods following the standard pediatric immunizations administered at 2 months of age. We included all infants who were born in Ontario between April 1, 1994 and March 31, 1996 (when the whole-cell pertussis vaccine was in use), and who had follow-up data available until at least 6 months of age. In Ontario, vaccination against pertussis was administered as part of a combination vaccine, which also includes diphtheria, tetanus, polio, and *Haemophilus influenza* type b. This pentavalent formulation was the only vaccine administered at 2 months of age in Ontario during the study period.

All study datasets were linked using unique encoded identifiers and analyzed at the ICES. ICES data includes Ontario residents covered by the publicly-funded Ontario Health Insurance Plan, encompassing virtually all people living in the province, but may exclude recent immigrants. Pediatric vaccinations were identified using physician billing claims data from the Ontario Health Insurance Plan database. To ascertain the 2-month vaccinations, we identified Ontario Health Insurance Plan billing codes for general vaccination occurring on the exact due date (61 days assuming an average month length of 30.5 days) as well as any vaccinations 14 days before and up to 40 days after the due date to allow for variations in scheduling. All-cause acute care hospital admissions were identified using the Canadian Institute for Health Information’s Discharge Abstract Database, and Ontario Health Insurance Plan billing data was used to ascertain all-cause ED visits during the study period.

### Data analysis

To conduct our analysis of ED visits or hospitalizations following immunization, we utilized the self-controlled case series design in which individual study subjects serve as their own control. In this design, the analysis only includes individuals who were both vaccinated and had an event of interest during the observation period. For each individual in our study, the index date for the exposure was the date of the 2-month pertussis vaccination. The follow-up time was then divided into three distinct intervals: an exposed period (or at-risk period), an unexposed period (or control period), and a washout period between the exposed and unexposed periods. Our selection of the at-risk and control periods was based on our previous studies of ED visits and/or hospitalizations following 2-, 4-, and 6-month immunizations, and our previous study comparing whole-cell and acellular pertussis vaccines. ^11,26^ For the 2-month vaccination, the at-risk period was 0 to 2 days following vaccination and the control period was 9 to 18 days post-vaccination. For infants with more than one vaccination in the database during the 2-month target period, the first vaccination was used as the index vaccination. If another vaccination occurred within the observation period (0 to 18 days after the index vaccination) for a given infant, then this individual was excluded from analysis. We calculated the relative incidence of ED visits and/or hospital admissions in the at-risk period versus the control period using a fixed-effects conditional Poisson regression model. By design, this type of regression model controls for non-time-varying individual-level characteristics, thereby allowing each individual to serve as his/her own control. To control for the dependence of multiple events occurring close together in time (e.g., an ED visit leading to an admission, or serial ED visits), each individual was classified as having “one or more events” or “no events” in each of the at-risk and control periods.

In order to determine whether the relative incidence of the composite outcome varied between males and females, we included an exposure time period by sex interaction term in the self-controlled case series conditional Poisson model. A likelihood ratio test was used to compare the full model including the interaction term to the reduced model without the interaction term in order to test whether the interaction term was statistically significant. ^30^ The parameter estimate from the interaction term can be exponentiated to yield a relative incidence ratio (RIR) which is equivalent to the ratio of relative incidence in females to the relative incidence in males: an intuitive measure of the magnitude of the difference in relative incidences for females versus males. To separate the effects of sex and birth weight, we also presented a model with adjustment for birth weight. Similarly, we tested a model that included an exposure time period by birth weight quintile interaction term in order to estimate the effect of lower birth weight on the relative incidence of events. This model was also adjusted for sex. This RIR has the added benefit of allowing us to overcome the impact of the ‘healthy vaccinee effect’, the decision by parents and health care providers to forgo vaccination when a child is acutely ill resulting in the administration of vaccines to children who are in a comparatively healthy state. ^28,31,32^ Such an effect results in a marked reduction in events prior to vaccination and can mask any adverse effect in the immediate post-vaccination period. Using the ratio of relative incidence in two subgroups (e.g., males and females) can largely cancel out the healthy vaccinee effect where it is expected to behave similarly in the two subgroups.

### Sensitivity analyses

We conducted several sensitivity analyses to evaluate the robustness of our conclusions. To estimate the effects of sex and birth weight on the risk of more severe events following immunization, we modeled the relative incidence of hospital admissions alone. Since we have previously observed that most adverse events following immunization occur within the first 24 hours, ^13^ we conducted a sensitivity analysis restricting the risk period to the first day post-vaccination. Lastly, to examine the effect of additional vaccine doses, we repeated our analysis for the 4- and 6-month vaccinations.

## Abbreviations

RIR: relative incidence ratio
CI: confidence interval
ED: Emergency Department
