# Supplementary material for: Effects of sex and birth weight on non-specific health services use following whole-cell pertussis vaccination: a self-controlled case series analysis
Source: Hum Vaccin Immunother. 2019 Apr 5;15(10):2399–404. doi: 10.1080/21645515.2019.1586029 (PMC6816393; doi:10.1080/21645515.2019.1586029)
Supplement: Supplemental Material [file khvi-15-10-1586029-s001.docx]

Supplementary Table 1. Emergency Department Visits and/or Hospitalization Following 2-Month Vaccination Overall and by sex and Birth Weight Quintiles, 1-day Risk Period

| **Sex** | **Vaccinated Children** | **Events During Risk Period**  **(Day 0)** | **Events During Control Period (Days 9-18)** | **Relative Incidence of Events**  **(95% CI)**  **Unadjusted** | **Relative Incidence**  **of Events**  **(95% CI)**  **Adjusted for bweight** | **Unadjusted Relative Incidence Ratio (95% CI), *P* value** | **Adjusted Relative Incidence Ratio (95% CI), *P* value** |
| --- | --- | --- | --- | --- | --- | --- | --- |
| Male | 106674 | 288 | 1798 | 1.44 (1.27, 1.63) | 2.54 (1.29, 5.01) | 1 (ref) | 1 (ref) |
| Female | 101510 | 218 | 1407 | 1.39 (1.21, 1.61) | 2.40 (1.25, 4.62) | 0.97 (0.80, 1.17), p= 0.7306 | 0.94 (0.78, 1.14), p=0.5632 |
| **Birth Weight Quintile** | **Vaccinated Children** | **Events During Risk Period**  **(Day 0)** | **Events During Control Period (Days 9-18)** | **Relative Incidence of Events**  **(95% CI)**  **Unadjusted** | **Relative Incidence**  **of Events**  **(95% CI)**  **Adjusted for sex** | **Unadjusted Relative Incidence Ratio (95% CI), *P* value ^1^** | **Adjusted Relative Incidence Ratio (95% CI), *P* value ^2^** |
| Q1: >=3871g | 40724 | 80 | 558 | 1.29 (1.02, 1.63) | 1.31 (1.03, 1.67) | 1 (ref) | 1 (ref) |
| Q2: 3581-3870g | 41031 | 90 | 636 | 1.27 (1.02, 1.59) | 1.30 (1.03, 1.64) | 0.99 (0.71, 1.36), p=0.9366 | 0.99 (0.72, 1.37),  p=0.9516 |
| Q3: 3341-3580g | 41596 | 93 | 622 | 1.35 (1.08, 1.67) | 1.38 (1.09, 1.74) | 1.04 (0.76, 1.44), p=0.7970 | 1.05 (0.76, 1.45),  p=0.7665 |
| Q4: 3063-3340g | 43129 | 122 | 683 | 1.61 (1.33, 1.95) | 1.65 (1.34, 2.04) | 1.25 (0.92, 1.69), p=0.1554 | 1.26 (0.93, 1.70), p=0.1417 |
| Q5: <=3062g | 41704 | 121 | 706 | 1.54 (1.27, 1.87) | 1.59 (1.28, 1.97) | 1.29 (1.02, 1.63), p= 0.0330 | 1.21 (0.89, 1.64),  p=0.2238 |

^1^ *P* value for overall interaction between birth weight and risk period (unadjusted model) = 0.3921

^2^ *P* value for overall interaction between birth weight and risk period (model adjusted for sex) = 0.3632

**Supplementary Figure Captions and Footnotes**

Supplementary Figure 1: Emergency Department Visits and Admissions on each day Relative to 2-Month Vaccination, by sex

Day 0 is date of vaccination

Horizontal axis: days pre- and post-vaccination

Vertical axis: Number of Emergency Department visits and hospitalizations

Supplementary Figure 2. Emergency Department Visits and Admissions on each day Relative to 2-Month Vaccination, by Birth Weight Quintile

Day 0 is date of vaccination

Horizontal axis: days pre- and post-vaccination

Vertical axis: Number of Emergency Department visits and hospitalizations
